# Recombinant expression library of *Pyrococcus furiosus* constructed by high-throughput cloning: a useful tool for functional and structural genomics

**DOI:** 10.3389/fmicb.2015.00943

**Published:** 2015-09-11

**Authors:** Hui Yuan, Li Peng, Zhong Han, Juan-Juan Xie, Xi-Peng Liu

**Affiliations:** State Key Laboratory of Microbial Metabolism, School of Life Sciences and Biotechnology, Shanghai Jiao Tong UniversityShanghai, China

**Keywords:** ligation-independent cloning, *Pyrococcus furiosus*, recombinant expression library, high-throughput cloning, phosphorothioate modification

## Abstract

Hyperthermophile *Pyrococcus furiosus* grows optimally near 100°C and is an important resource of many industrial and molecular biological enzymes. To study the structure and function of *P. furiosus* proteins at whole genome level, we constructed expression plasmids of each *P. furiosus* gene using a ligase-independent cloning method, which was based on amplifying target gene and vector by PCR using phosphorothioate-modified primers and digesting PCR products by λ exonuclease. Our cloning method had a positive clone percentage of ≥ 80% in 96-well plate cloning format. Small-scale expression experiment showed that 55 out of 80 genes were efficiently expressed in *Escherichia coli* Strain Rosetta 2(DE3)pLysS. In summary, this recombinant expression library of *P. furiosus* provides a platform for functional and structural studies, as well as developing novel industrial enzymes. Our cloning scheme is adaptable to constructing recombinant expression library of other sequenced organisms.

## Introduction

Some extreme environments stretch well beyond those considered optimal for common life form, such as relative extremes of temperature, pH, salinity, pressure, and radiation. Microorganisms living in these environments are called extremophiles, which include (hyper)thermophiles, psychrophiles, alkaliphiles, acidophiles, and halophiles (Cavicchioli et al., 2011). Extremophiles are valuable model organisms for understanding the physical limits to life occurring, and the origins of life (Cavicchioli, 2002; Cavicchioli et al., 2011). Extremophiles are also significant natural resources for discovering novel enzymes and other bioactive compounds used in industry and pharmaceuticals. Many useful enzymes have been isolated from these extremophiles. Generally, these proteins show unique features consistent with their origins, such as extreme thermostability and resistance against chemical denaturants, organic solvents, high salinity, and extremes of pH (Fujinami and Fujisawa, 2010; Wende et al., 2010; Fuciños et al., 2012; Sharma et al., 2012; Purohit and Singh, 2013). Many industrial enzymes isolated from (hyper)thermophiles, such as DNA polymerase, amylases, cellulases, esterases, and proteases, are now used in life sciences, food, chemical, and pharmaceutical industries and environmental biotechnology (Lu and Erickson, 1997; Harris et al., 2001; Hogrefe et al., 2002; Van den Burg, 2003; Yang et al., 2007; Cavicchioli et al., 2011). The genus *Pyrococcus* is a group of obligate anaerobic hyperthermophilic archaea that grow optimally near 100°C (Fiala and Stetter, 1986). As the first isolated *Pyrococcus* species, *Pyrococcus furiosus* (*P. furiosus*) is well-characterized and its genomic DNA sequencing has been completed (Robb et al., 2001). Because of the advantage of thermostability several common industrial enzymes have been characterized and used in biotechnology (Harris et al., 2001; Verhagen et al., 2001; Van den Burg, 2003; Yang et al., 2004, 2007). Many “-omics”-based approaches, including transcriptomics (Schut et al., 2003; Yoon et al., 2011), comparative genomics (Lecompte et al., 2001), proteomics (Menon et al., 2009), and metallomics (Cvetkovic et al., 2010), have been used to study the physiological feature of *P. furious*, but determining the biochemical properties and crystal structures of *P. furiosus* proteins are necessary to fully understand gene functions.

Structural genomics, along with the genomics, focuses on determining protein structures on the scale of the whole genome (Adams et al., 2003; Joachimiak, 2009). *P. furiosus* is a model archaea in structural genomics studies. Until now, more than two hundred protein structures from *P. furiosus* have been released in PDB (http://www.rcsb.org/pdb). These structures are from proteins involved in almost each cellular metabolism pathway, especially in the genetic information process (Nakamura et al., 2008; Williams et al., 2008; Klein et al., 2011; Kim et al., 2012; Lapinaite et al., 2013; Ramia et al., 2014), and contribute our understanding to the enzymatic catalytic mechanism. However, there is still a need to obtain more biochemical and structural knowledge of proteins/enzymes to further understand the physiological features of *P. furiosus*.

Restriction enzyme cloning plays an important role in preparing recombinant proteins for biochemical characterization and structural studies. However, it is not suitable for investigating protein's function and structure in high throughout. To clone genes in high throughout format, several cloning methods have been developed, such as ligation-independent cloning (LIC) (Aslanidis and de Jong, 1990; Nisson et al., 1991; Yang et al., 1993; Kaluz and Flint, 1994; Tseng, 1999; Coljee et al., 2000; Donahue et al., 2002; Blanusa et al., 2010), and hybridization cloning (Tillett and Neilan, 1999; Van den Ent and Löwe, 2006). These high throughout cloning techniques are independent of restriction endonuclease and are based on universal treatment of insert and vector DNA. We constructed a *P. furiosus* recombinant expression library using phosphorothioate primers and λ exonuclease-based LIC method we developed previously (Liu and Liu, 2010). Cloning of 2125 *P. furiosus* genes were completed by a three-person team in 3 weeks. This *P. furiosus* recombinant expression library also provides a repository for studying thermostable enzymes and protein crystal structures.

## Materials and methods

### Materials

T4 polynucleotide kinase and *Taq* DNA polymerase were purchased from Fermentas (Shanghai, China), and *KOD*-plus DNA polymerase was obtained from Toyobo (Shanghai, China). The recombinant λ exonuclease was prepared as described (Liu and Liu, 2010). pDEST17 vector and all primers used in this study were purchased from Invitrogen (Shanghai, China). *P. furiosus* genomic DNA was purchased from ATCC (Manassas, USA). *Escherichia coli* (*E. coli*) strain DH5α was used in gene cloning and Rosetta 2(DE3)pLysS strain (Massachusetts, USA) was used to express *P. furiosus* protein. All other reagents were of analytical grade.

### Amplification of target gene and linear vector by PCR

*KOD*-plus DNA polymerase was used for amplification of *P. furiosus* genes and the pDEST17 vector. 5′-phosphorylation reaction of phosphorothioate-modified common primers was performed for 2 h at 37°C as follows: 10 nmole primers, 50 U T4 polynucleotide kinase and 200 nmol ATP in T4 PNK reaction buffer. The phosphorylated primers were used directly without any purification. All experiments, including PCR, recombinant reaction, and transformation and screening positive clones, were performed in 96-well plate. Each *P. furiosus* gene fragment was amplified by a two-step PCR using gene-specific (Table S1 in the Supplementary Material) and 5′-phosphorylated phosphorothioate-modified common primers of Common-gene-F (5′*CAAAAAAGCA**GGCT*C-C-C-A-TATG 3′) and Common-gene-R (5′*CAAGAAAGCTG GGT*C-G-G-A-TCCACTAGT 3′). Symbols of – denote the phosphorothioate modification. The complementary sequences between gene-specific and common primer are underlined. The homologous sequence (for generating the complementary 3′ overhang) between insert and vector is shown in italic. First-step PCR reaction (5 μl) was prepared in 1 × PCR buffer and consisted of 1 ng of *P. furiosus* genomic DNA, 0.2 μM gene-specific primers, 200 μM dNTPs, 2.0 mM MgSO_4_, and 0.05 U *KOD*-plus DNA polymerase. After capping with 30 μl liquid paraffin, first-step PCR was subjected to predenaturation at 98°C for 3 min, 15 cycles of 95°C for 30 s, 50–60°C (based on the Tm of specific primers) for 30 s and 72°C for 1–3 min (1 min/1 kb length), followed by a 3 min extension at 72°C. Then, the first-step PCR mixtures were directly diluted into 25 μl common PCR reaction mixtures consisting of 1 × PCR buffer, 0.3 μM 5′-phosphorylated phosphorothioate-modified common primers, 240 μM dNTPs, 2.0 mM MgSO_4_, and 0.3 U *KOD*-plus DNA polymerase. The second-step PCR was performed by predenaturation at 98°C for 3 min, 20 cycles of 95°C for 30 s, 50°C for 30 s, and 72°C for 1–3 min, followed by a 3 min extension at 72°C. The linear prokaryotic expression vector pDEST17 (about 4.3 kb) was amplified by PCR using 5′-phosphorylated phosphorothioate-modified primers of common-pDEST17-F (5′*AGCCTGCTTTTTTG*T-A-C-A-AACTTGTT 3′) and common-pDEST17-R (5′*ACCCAGCTTTCTTG*T-A-C-A-AAGTG 3′). The PCR reaction (50 μl) was carried out using 5 ng of circular pDEST17 vector DNA and 0.4 μM primers with the following condition: predenaturation at 98°C for 3 min, 20 cycles of 95°C for 30 s, 52°C for 30 s, and 72°C for 5 min, and a final extension at 72°C for 8 min.

### Recombination reaction of insert and vector DNA

The PCR products of *P. furiosus* genes and linear pDEST17 vector were directly used in the next step of λ exonuclease digestion reaction without any purification. Treatment of DNA fragments with λ exonuclease was performed using 96-well plates. Exonuclease digestion was performed at 37°C for 5 min in a volume of 10 μl containing 66 mM glycine-NaOH (pH 9.4), 50 ng λ exonuclease, 200 ng protease K, 5′-phosphorylated and phosphorothioated gene fragment (100–300 ng) and linear pDEST17 vector (50 ng).

### Transformation of recombinant plasmid and identification of positive clone

Transformation of *E. coli* DH5α competent cell and identification of positive recombinant plasmid by colony PCR were performed on a scale of 96-well plate. The exonuclease-treated DNA mixtures (2 μl) were directly transformed into 25 μl of competent *E. coli* DH5α cells according to standard transformation protocol. After heat shock at 42°C for 1 min, 100 μl of LB was added into bacteria culture, and then incubated at 37°C for 30–60 min. One third of transformed bacteria were plated on LB solid culture media containing 100 μg/ml ampicillin. For each gene, two clones were picked for screening the presence of the insert by colony PCR using *Taq* DNA polymerase. Colony PCR reactions (15 μl) consisted of 1 × PCR buffer, 0.3 μM specific primers, 200 μM dNTPs, 2.0 mM MgSO_4_, approximately 0.5 μl bacteria culture, and 1 U *Taq* DNA polymerase. Amplification reaction was carried out using the following condition: predenaturation at 95°C for 10 min, 30 cycles of 95°C for 30 s, 60°C for 30 s, and 72°C for 1–3 min, and a final extension at 72°C for 3 min.

### Expression of *P. furiosus* proteins in small scale

To check the induced expression of *P. furiosus* recombinant proteins, the expression plasmids were transformed into *E. coli* Rosetta 2(DE3)pLysS expression host. One clone was picked into 3 ml LB media containing 34 μg/ml chloramphenicol and 100 μg/ml ampicillin, and cultured at 37°C overnight. The overnight cultures were diluted into fresh media in a volume ratio of 1:5. After refreshing of the diluted bacteria for 15 min, isopropy-β-D-thiogalactoside (IPTG) was added into the cultures with a final concentration of 0.5 mM to induce the expression of *P. furiosus* recombinant protein at 37°C for 3 h. The bacteria cultures (1 ml) were harvested by centrifugation, resuspended in 1 × protein loading buffer (100 μl), and boiled for 10 min at 95°C. The bacterial lysates were subjected to 15% SDS-PAGE to confirm the expression of *P. furiosus* recombinant proteins.

## Results

### High throughout cloning of *P. furiosus* genes

**Figure 1:**
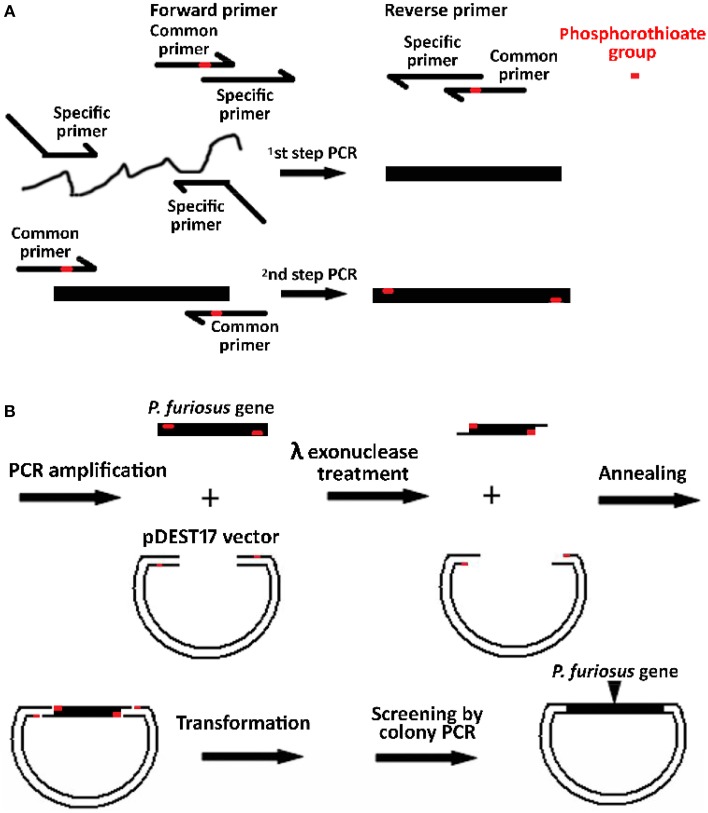
**Schematic representation for constructing of a *P. furiosus* recombinant expression library. (A)** Amplification of *P. furiosus* genes. Each *P. furiosus* gene fragment was prepared by a two-step amplification reaction using gene-specific primers and the 5′-phosphalated and phosphorothioate-modified common primers. The 3′ sequences (12 bases) of common primers are complementary to the 5′ sequences (12 bases) of the first-step PCR fragment. The final PCR-amplified fragments contain four successive phosphorothioate groups (shown in red) near the 5′ end. **(B)** Recombination between pDEST17 vector and *P. furiosus* gene fragments. The 5′-phosphalated and phosphorothioate-modified gene fragment and the linear vector were directly treated by λ exonuclease without any purification, and the phosphorothioate groups block the digestion of exonuclease and result in the generation of 3′ overhangs that are complementary between gene fragment and vector. Finally, the annealed plasmid was repaired *in vivo* by *E. coli* after transformation.

The schematic presentation of *P. furiosus* genes by high throughput (HTP) cloning is shown in Figure 1. Each *P. furiosus* gene fragment was amplified by a two-step PCR and contained four successive phosphorothioate groups at each terminus (shown in red). Prokaryotic recombinant expression vector pDEST17 was chosen to express *P. furiosus* protein. The PCR-amplified linear pDEST17 vector fragment also contained four successive phosphorothioate groups at each terminus. As shown in Figure 1B, phosphorothioates can strongly hinder the processive hydrolysis of double-stranded DNA from 5′ terminus by λ exonuclease (Nikiforov et al., 1994), and result in a 15 nt 3′ overhang. The base sequences of 3′ overhangs are complementary between insert and vector, and form a circular recombinant plasmid with four nicks. The nick in the circular recombinant plasmid resulting from combining the two modified DNAs can be repaired by bacterial DNA repair system after transforming into *E. coli* cells.

### Protease K remarkably increases cloning efficiency

**Figure 2:**
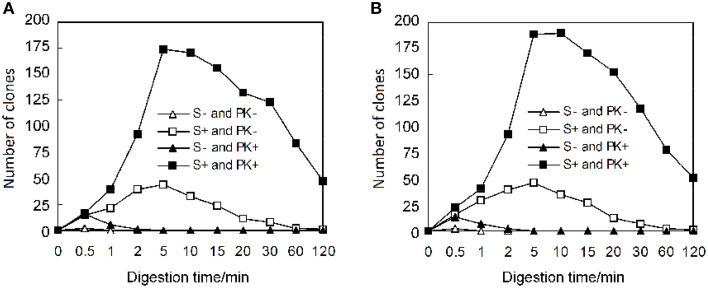
**Promotion of cloning efficiency by protease K**. The mixtures of PCR fragments (50 ng pDEST17 and 50 ng PF1415) were incubated with 50 ng λ exonuclease at 37°C for various times in 10 μl reaction volume before transforming into *E. coli* DH5α. For the mixtures of unpurified PCR fragments **(A)**, 200 ng protease K was firstly added into the mixtures of PCR fragments and incubated for 5 min before the digestion of DNA by λ exonuclease. However, for the purified PCR fragments **(B)**, the digestion of DNA was carried out on simultaneous addition of λ exonuclease and protease K. After λ exonuclease digestion, 3.3 μl of the reaction mixtures were used to transform *E. coli* DH5α competent cells. The abbreviations of S and PK indicate phosphorothioate and protease K.

When λ exonuclease generated a 3′-overhang DNA, the remaining *KOD*-plus DNA polymerase in PCR reaction possibly degraded the 3′-overhang, which would decrease the number of intact 3′-overhangs and resulted in less recombinant clones. Considering that protease K can be used to degrade DNA polymerase, we tested the effect of protease K on cloning efficiency. Consistent with the analysis, protease K remarkably increased the number of recombinant clones (Figure 2A). If the digestion time was longer than 10 min, the clone number decreased in the absence of protease K. However, the cloning efficiency did not noticeably change between 5 and 30 min in the presence of protease K; even some clones were generated after a 120 min treatment by λ exonuclease. Considering that protease K also degrades the λ exonuclease, the enzyme may also be harmful for the digestion of PCR fragments by λ exonuclease. We further studied the effect of protease K using purified PCR fragments free of DNA polymerase. Instead of decreasing cloning efficiency, the protease K can also increase the number of recombinant clones, especially under extended treatment with λ exonuclease (Figure 2B). Moreover, the purification of PCR fragments did not result in a distinct increase of clone numbers (Figure 2), indicating that the other elements of PCR mixtures were not harmful to the cloning efficiency. The possible role of protease K in promoting generation of 3′-overhang DNA is discussed later.

### Cloning of *P. furiosus* genes at whole genome level

Before starting to clone genes in 96-well plates, we first determined the cloning efficiency of various length *P. furiosus* genes. Considering that the insert was excessive to vector, the effect of ratio of insert to vector was less significant. In general the number of recombinant clones was inversely in proportion to the length of cloned genes (Table 1). For the inserts shorter than 2500 bp, the number of recombinants was about 50–400. For DNA inserts longer than 2500 bp, the number of recombinants sharply decreased. The longest *P. furiosus* gene PF0440 (4.6 kb in length, with a ratio of insert to vector 2) only generated 7 clones. To compare the cloning efficiency of different length DNA fragments, the molar ratios of insert to vector is calculated for each gene (Table 1). Interestingly, for the insert PF0622 with the highest ratio of insert to vector, the clone number was not the highest. It may be that the DNA termini number of PF0622 had become a limit to the molecule number of λ exonuclease, and that this resulted in the decrease of insert with two intact 3′-overhangs. In summary, the cloning efficiency of our HTP cloning method was suitable for constructing a *P. furiosus* recombinant expression library.

**Table 1:** **Number of recombinant clones of *P. furiosus* genes of various length**.

**ORFs**	**Gene length/bp**	**Molecule ratio of gene to vector**	**Number of recombinants**
PF0622	99	36.6	176
PF0741	300	15.2	351
PF1415	999	12.1	189
PF0205	1506	8.7	121
PF1521	2001	6.2	97
PF0201	2493	5.8	63
PF1983	2988	4.3	41
PF0495	3642	3.4	23
PF0287	4194	2.7	12
PF0440	5220	2.1	7

*Two microliter (100–300 ng) gene fragment and 1 μl (50 ng, ≈ 0.017 pmol) linear pDEST17 fragment were mixed into 10 μl reaction and treated with 50 ng λ exonuclease for 5 min at 37°C. After λ exonuclease digestion, 3.3 μl the reaction mixtures were used to transform E. coli DH5α competent cells, whose transformation efficiency is about 3.2 × 10^5^/μg circular pUC18 vector*.

**Figure 3:**
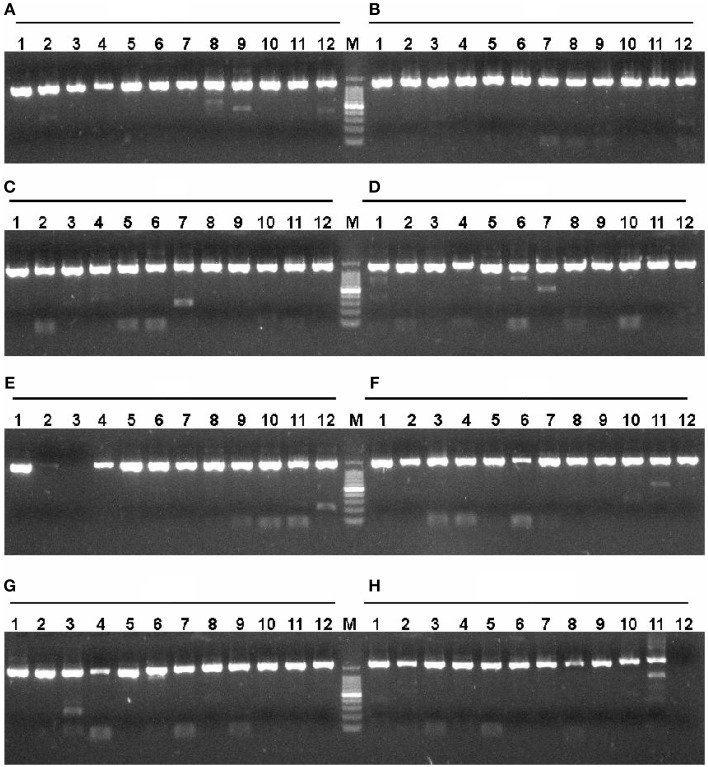
**Amplification of *P. furiosus* genes using two-step PCR**. Each *P. furiosus* gene fragment was amplified by a two-step PCR using gene-specific and common primers according to the description in Materials and Methods Section. The annealing temperature was 54°C, and the extension time was 2.5 min. Each lane corresponds to a gene of the 17th microtiter plate in Tables S1, S2 in the Supplementary Material. The labels **(A–H)** mean the respective rows and the Arabic numbers mean the specific columns in the 96-well plate. Therefore, each individual combination of row and column represents a specific gene in the 96-well plate. The symbol M denotes the 100 bp DNA marker (100, 200, 300, 400, 500, 600, 700, 800, 900, 1000, and 1500 bp).

**Figure 4:**
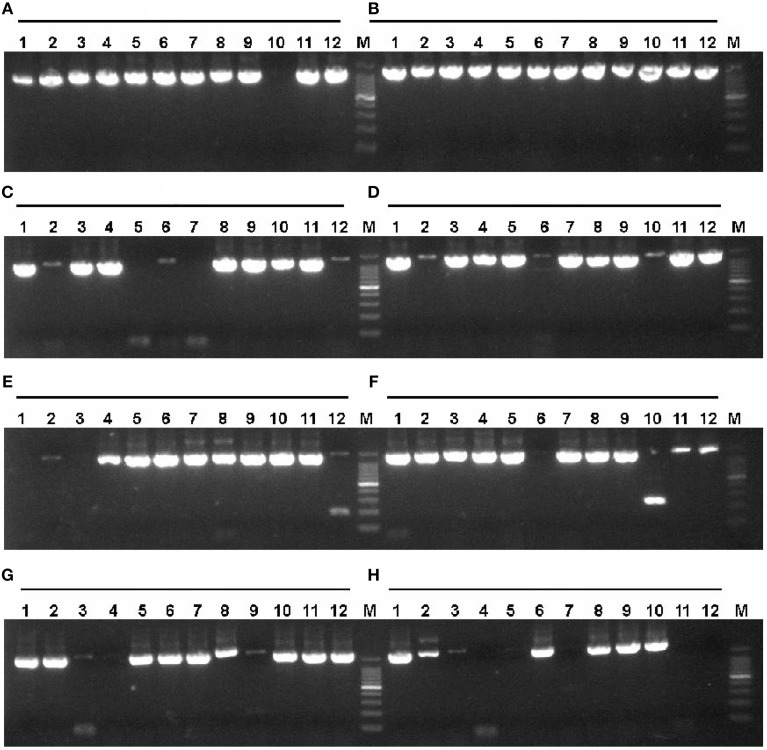
**Colony PCR of *P. furiosus* genes to identify positive clones**. Colony PCR reactions (15 μl) were performed according to the description in Materials and Methods Section. The annealing temperature was 54°C, and the extension time was 2.5 min. Each lane corresponds to the gene of the 17th plate in Tables S1, S2 in the Supplementary Material. The labels **(A–H)** mean the respective rows and the Arabic numbers mean the specific column in the 96-well plate. Therefore, each individual combination of row and column represents a specific gene in the 96-well plate. The symbol M denotes the 100 bp DNA marker (100, 200, 300, 400, 500, 600, 700, 800, 900, 1000, and 1500 bp).

According to the described protocol, 2125 ORFs were amplified from *P. furiosus* by two-step PCR. To be most efficient in amplifying genes by PCR, all *P. furiosus* ORFs were classified into 22 groups (95 ORFs each group, Table S1 in the Supplementary Material) according to gene length and Tm values of primers. The amplified DNA fragments of the 17th group genes (the 17th plate) were checked by 1% agarose-gel electrophoresis (Figure 3). Agarose-gel electrophoresis images of other gene groups are available on request. Using the λ exonuclease cloning method, more than 80% of all *P. furiosus* genes were successfully cloned in the first cloning experiment. Colony PCR results of the 17th group genes are shown in Figure 4. The colony PCR electrophoresis images of other groups are available on request. Previous research reported that primer dimers were harmful for cloning of real gene fragments by Gateway cloning (Hartley et al., 2000). We tested if primer dimers were the reason for the failure of some genes′ cloning in our method. After removal of primer dimers by agarose-gel electrophoresis, more than 80% of failed genes were successfully cloned except for 56 genes. Checked by concentration assay, all the 56 genes′ PCR products were lower than 10 ng/μl. On optimizing the PCR amplification conditions, 46 out of the 56 remaining genes were amplified in high yield and successfully cloned. Finally, the *P. furiosus* recombinant expression library (Table S2 in the Supplementary Material) was successfully constructed except for 10 genes (PF0012, PF0064, PF0075, PF0143, PF0504, PF0562, PF0765, PF0785, PF0966, and PF1120; highlighted in Table S2 in the Supplementary Material).

### Recombinant expression of *P. furiosus* proteins

**Figure 5:**
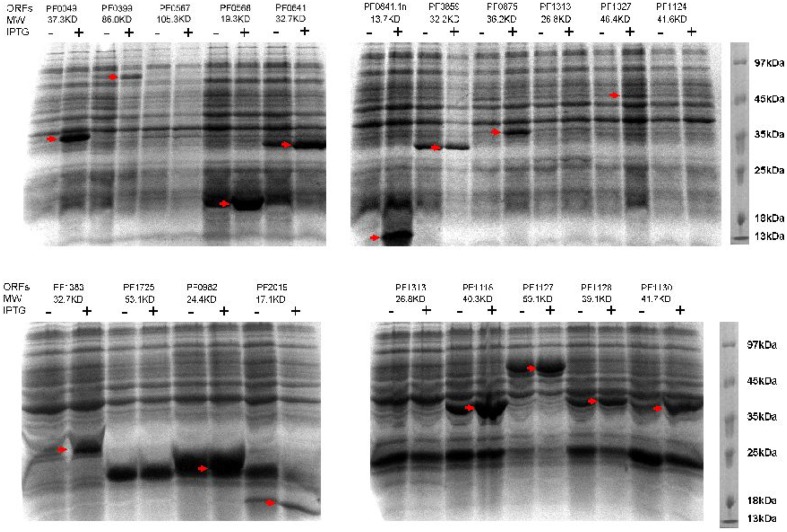
**Induced expressions of *P. furiosus* genes in Rosetta 2(DE3)pLysS cells**. Twenty *P. furiosus* expression plasmids were randomly selected for recombinant expression in *E. coli* Rosetta 2(DE3)pLysS cells. The expression was induced with 0.5 mM final concentration of IPTG. The ORF numbers and the molecular mass of their proteins are listed at the top of each gel image. The expressed *P. furiosus* proteins are marked by red arrows.

To verify the feasibility of heterologous expression of *P. furiosus* proteins in *E. coli*, we firstly selected 20 recombinant plasmids (including the positive control of PF2019 that was successfully expressed in our lab.) to examine their expression level in the *E. coli* Rosetta 2(DE3)pLysS strain. Fifteen out of 20 recombinant proteins (75%) could be efficiently expressed in the *E. coli* host upon IPTG induction (Figure 5). In addition to the 20 proteins in Figure 5, we further checked the inductive expression of another 60 *P. furiosus* recombinant proteins in Rosetta 2(DE3)pLysS cell. Forty recombinant proteins were successfully expressed (Figure S1 in the Supplementary Material). These results showed that our recombinant expression library could be used to express *P. furiosus* protein efficiently.

## Discussion

In comparison to other LIC cloning technologies, our method has a cloning efficiency at least 10 times higher (Liu and Liu, 2010). Otherwise the successive phosphorothioates strongly inhibit the λ exonuclease digestion reaction; so that it can bear some relaxed operation conditions, such as flexible reaction time, variable amounts of DNA fragments, which make the method more plausible for HTP cloning (Quan and Tian, 2011).

Why can the protease K improve the cloning efficiency in our exonuclease-based LIC? It may be that protease K degrades the excessive λ exonuclease, and thus may protect the insert and vector DNAs from being hydrolyzed excessively by λ exonuclease. In comparison to the natural phosphodiester bond, the phosphorothioate group can largely decelerate the hydrolysis of phosphodiester bond by λ exonuclease, and results in the pause of λ exonuclease ahead of a phosphorothioate group. The protease K will degrade the paused λ exonuclease and terminate the λ exonuclease's digestion of double-stranded DNA ahead of phosphorothioate. This role of protease K on degrading the paused exonuclease is similar to its degrading of the DNA polymerase that encounters and stagnates on a DNA mismatch in allele-specific primer extension (Hultin et al., 2005).

Characterizing biochemical properties and crystal structures of *P. furiosus* proteins will help understand the molecular basis of *P. furiosus's* adaption to high temperature and the catalytic mechanisms of its extremely thermostable enzymes. Our primary results on IPTG-induced expression of 80 *P. furiosus* recombinant proteins demonstrated that about 70% of proteins could be produced in *E. coli* Rosetta 2(DE3)pLysS strain (Figure 5 and Figure S1 in the Supplementary Material). The possible causes for failed expression include the frameshift mutations in the coding regions, and inappropriate selection for vector and expression host. After examining the DNA sequences of the clones not expressing the recombinant proteins, we found some of them have frameshift mutations in the coding regions, and resulted mainly from the DNA sequences provided by PCR primers. This phenomenon was also reported by other researchers (Li et al., 2011), and the percentage of error primers is about 12% in our study (data not shown). On the other hands, for the unexpressed proteins with a correct coding sequence, it is also possible to improve the recombinant expression of *P. furiosus* proteins by changing the expression vector and host.

Previously, many recombinant proteins from *P. furiosus* have been biochemically characterized (Kiyonari et al., 2009; Ishino et al., 2013; Yuan et al., 2013; Shiraishi et al., 2015). Many structures of *P. furiosus* protein have been resolved even a small portion of them were published (Adams et al., 2003; Wang et al., 2005). These structural studies involved proteins responsible for processing the double-stranded end in homologous recombination, CRISPR, transcription, and polysaccharide hydrolysis (Nakamura et al., 2008; Williams et al., 2008; Klein et al., 2011; Kim et al., 2012; Lapinaite et al., 2013; Ramia et al., 2014). Using our expression library, the crystallographic analyses of some proteins involved in genetic information processing are now in progress to further understand the structure and functions of nucleic acid metabolism in *P. furiosus*.

In summary, our expression library provides a platform for studying the biochemical characteristics and crystal structures of *P. furiosus* proteins, as well as developing new enzymes for use in industry and scientific research. Furthermore, our method is very useful and convenient for inserting a DNA fragment into a vector, it is also practical to construct recombinant expression library of other model organisms, or to test the recombinant expression of one protein by inserting its gene into different expression vectors, and determining the optimal expression vector for a specific recombinant protein.

### Conflict of interest statement

The authors declare that the research was conducted in the absence of any commercial or financial relationships that could be construed as a potential conflict of interest.
