# Supplementary material for: Recombinant expression library of Pyrococcus furiosus constructed by high-throughput cloning: a useful tool for functional and structural genomics
Source: Front Microbiol. 2015 Sep 11;6:943. doi: 10.3389/fmicb.2015.00943 (PMC4566052; doi:10.3389/fmicb.2015.00943)
Supplement: Supplementary file 3 [file DataSheet1.DOC]

**Supplementary Material**

**Title of Paper:**

Recombinant expression library of *P. furiosus* constructed by high-throughput cloning: a useful tool for functional and structural genomics

**Author names:**

Hui Yuan^†^, Li Peng^†^, Zhong Han^†^, Juan-Juan Xie, Xi-Peng Liu*

**Affiliation of Authors:**

State Key Laboratory of Microbial Metabolism, and School of Life Sciences & Biotechnology, Shanghai Jiao Tong University, 800 Dong-Chuan Road, Shanghai 200240, China

† These authors have contributed equally to this work.

* **E-mail address of the Corresponding Author:**

[xpliu@sjtu.edu.cn](mailto:xpliu@sjtu.edu.cn)

**Table S1 Base sequences of the primers used for amplifying *P. furiosus* genes**

All *P. furiosus* ORFs were classified into 23 groups based on their gene length and the Tm value of each pair of primer. The Tm value of primer, the gene length, and the location of primers on plate were listed.

**Table S2 Location of *P. furiosus* genes in 96-well plate**

The ORF numbers of each *P. furiosus* gene were listed. The highlighted genes were not successfully cloned into pDEST17 expression vector.

**Figure S1 The expression screening of partial *P. furiosus* recombinant proteins**

The recombinant *P. furiosus* proteins were randomly selected to express by induction with 0.5mM IPTG in 3ml culture. 15% SDS-PAGE was used to confirm the expression level of each *P. furiosus* recombinant protein. The ORFs were listed at the top of each gel image.
