# Supplementary figures and images for: Recombinant expression library of Pyrococcus furiosus constructed by high-throughput cloning: a useful tool for functional and structural genomics
Source: Front Microbiol. 2015 Sep 11;6:943. doi: 10.3389/fmicb.2015.00943 (PMC4566052; doi:10.3389/fmicb.2015.00943)

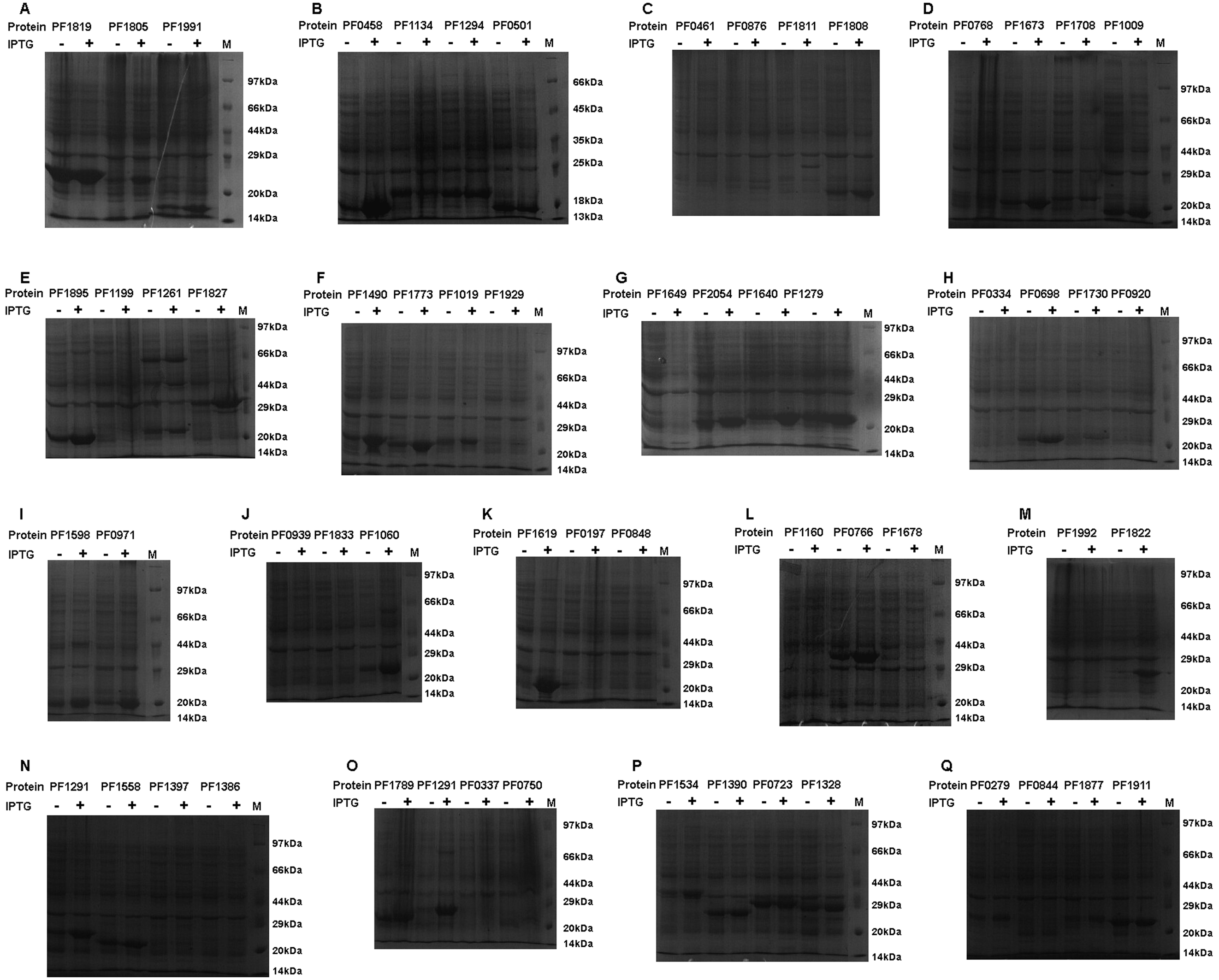

Supplement: Supplementary file 4 [file Image1.TIF]
